# When to mob? plasticity of antipredator behavior in common ravens’ families (*Corvus corax*) across offspring development

**DOI:** 10.1007/s10071-025-01976-9

**Published:** 2025-07-03

**Authors:** Silvia Damini, Christian R. Blum, Petra Sumasgutner, Thomas Bugnyar

**Affiliations:** 1 Department of Behavioral & Cognitive Biology, Faculty of Life Sciences, University of Vienna, Vienna, Austria; 2 Konrad Lorenz Research Center for Behavior and Cognition, Core Facility, Faculty of Life Sciences, University of Vienna, Vienna, Austria; 3 Haidlhof Research Station, University of Vienna and University of Veterinary Medicine Vienna, Vienna, Austria

**Keywords:** Antipredator behavior, Mobbing, Development, Ravens, Teaching

## Abstract

The ability to respond appropriately to predators is essential for survival. Because response options vary with predation context, anti-predator behavior is often flexible, context dependent and shaped by learning. Corvids engage in predator mobbing, which contains a vocal component (scolding) and predator-directed behaviors (approaches, attacks). Individuals typically gang up for mobbing and pass on information about predators; yet their expression of antipredator behavior is influenced by factors such as social status, age, and rearing conditions. Here we investigated the development of antipredator behavior in ravens, specifically the onset of mobbing and the extent to which these responses are affected by parental agitation. We exposed 12 captive families to a potentially dangerous human (DH) at two stages of offspring development: shortly after fledging and near independence. We tested the hypotheses that (i) parents are more protective when the offspring are young and that (ii) offspring show more predator-directed behaviors with increasing age. We found that (i) adults mobbed significantly more during the early test period and (ii) offspring were less likely to ignore the DH and showed increased engagement during the late test period. These findings suggest that parental anti-predator investment diminishes as offspring develop greater motoric and cognitive abilities. This reduced investment may encourage offspring to independently assess and respond to threats. Yet, they hardly engage in mobbing while they are with their parents. Future studies may clarify if the increase in offsprings’ interindividual variance in both mobbing components are indicative for the emergence of individuality.

**Supplementary Information:**

The online version contains supplementary material available at 10.1007/s10071-025-01976-9.

## Introduction

The ability to respond correctly to predators is essential for survival. The suite of behaviors aimed at detecting and avoiding predation is called antipredator behavior (Caro 2005). Performing antipredator behavior can be risky, and diminishes the time and energy that an individual has to increase its fitness by engaging in other adaptive behaviors, such as reproduction and foraging (Montgomerie and Weatherhead 1988; Lima and Dill, 1990, Clark 1994). Adaptive antipredator responses are under strong selection to diminish the impact of antipredator behavior on general fitness (Ives and Dobson 1987). Because response options can be varied, anti-predator behavior is often flexible, context dependent, and subject to learning.

To study antipredator behavior, it is essential to understand the ways in which flexibility is expressed. Animals can adapt their responses to varying levels of risk. For example, both Tufted titmice (*Baeolophus bicolor*) and Black-capped chickadees (*Poecile atricapillus*) assess predator risk based on predator size, adjusting their alarm calls and behaviors accordingly (Templeton et al. 2005; Courter and Ritchison 2010). Similarly, Siberian jays (*Perisoreus infaustus*) adjust their nest visitation patterns in response to predator activity, timing chick feeding to coincide with periods of lower predator presence (Eggers et al., 2005). While this flexibility spans the full range of antipredator behaviors, some behaviors appear to be more adaptable than others.

Mobbing behavior is a flexible antipredator strategy that includes harsh alarm vocalizations directed at the predator, referred to as ‘scolding’, and physical approaches to the predator (Altmann 1956). Scolding serves multiple functions: it can harass the predator into leaving, recruit conspecifics to aid mobbing (Curio 1978), and potentially signal male quality and social status (Ellis 2009). Group composition strongly influences mobbing intensity. For example, males of various species increase their mobbing intensity in the presence of females (da Cunha et al. 2017a). Similarly, Siberian jays show nepotistic antipredator protection, with kin-based groups mobbing predators more, and dominant males mobbing more intensively, particularly when their offspring are present (Griesser and Ekman 2005). Another aspect of mobbing flexibility lies in its informational role. Scolding can provide inexperienced individuals with crucial information about dangerous heterospecifics (Curio, Ernst & Vieth, 1978), allowing them to identify threats without direct exposure (Carlson and Griesser 2022). This form of social learning is particularly beneficial for young animals, who face higher predation risks due to their physical limitations and inexperience (Carlson and Griesser 2022).

Juveniles exhibit distinct antipredator responses that differ from adult behaviors, often emphasizing avoidance rather than direct engagement. For example, nestlings of Japanese tit (*Parus minor*) flee their nests upon hearing nest-specific scolding calls (Suzuki 2011), or crouch to hide themselves (Ha et al. 2020), while Easter bluebirds (*Sialia sialis*) become silent (Grabarczyk and Ritchison 2015). These are all behaviors directed away from the predator; but after observing adults mob, juveniles can also start to engage in mobbing themselves (Griesser and Suzuki 2016). These behaviors minimize predation risk but do not involve active mobbing. Over time, juveniles may transition to mobbing behaviors after observing adults (Carlson et al. 2020; Buitron, 1983). This developmental shift might reflect the high cost of mobbing for juveniles, who are less coordinated and more vulnerable than adults (Carlson and Griesser 2022). Before fully participating in mobbing, juveniles must learn to recognize predators (Carlson, Healy, & Templeton, 2020; Griesser and Suzuki 2016; Griesser and Suzuki 2017) and practice safe mobbing strategies (Carlson et al. 2020). The ability to adjust mobbing behavior based on social context, developmental stage, and experience highlights the flexibility of antipredator strategies and their critical role in survival.

Various studies have used a masked human as artificial predator (dangerous human) to elicit antipredator behavior in different species (e.g., Levey et al. 2009; Davidson et al. 2015). In corvids, this method has been used in particular to elicit mobbing. In a series of experiments, Marzluff and his team (Marzluff et al. 2010; Cornell et al. 2012; Swift and Marzluff 2015) showed that American crows (*Corvus brachyrhynchos*) can learn about novel predators and remember them for long periods of time. In the first study, crows learned to distinguish between a masked, dangerous human that caught and ringed them and a neutral human that did not (Marzluff et al. 2010). This happened through direct interaction with the dangerous human but also through horizontal social learning. One single experience was enough for learning to differentiate between the threatening and the neutral stimuli, and birds showed long term memory retention (up to five years). The second study provided experimental evidence also for vertical social learning (Cornell et al. 2012), as individuals that weren’t present during the original interactions with the dangerous mask produced alarm call when confronted with it. A follow-up study modified the procedure by exposing the crows to a masked human carrying a dead crow (Swift and Marzluff 2015). The crows reacted by performing alarm calls and avoiding the area where the experiment took place for at least 7 weeks. All those studies have been performed on dynamically formed groups composed of sub-adult and adult individuals, without specifically focusing on adults when they are taking care of their offspring. Using a similar design to Marzluff studies, Davidson et al. (2015) focused on wild jackdaws (*Corvus monedula*) with offspring: as expected, parents responded defensively towards masked humans previously associated to nestling manipulation by guarding their chicks from the perceived threat.

The antipredator behavior of the offspring has also been investigated in corvids but remains understudied compared to that of the adults. For instance, offspring of Black billed magpies (*Aphelocoma coerulescens*) and Florida scrub jays (*Aphelocoma coerulescens*) have been reported to observe others mobbing (Buitron, 1983; Francis et al. 1989) and to learn to recognize novel predators from their parents (Swift and Marzluff 2015). In Siberian jays, some juveniles have been observed mobbing soon after leaving the family but only after they have witnessed their parents mobbing (Griesser and Suzuki 2016). So, we know that the family phase seems to be critical for the antipredator behavior exhibited later in life, but we know little about the development of the antipredator behavior of the offspring while they are still in the family. In particular, observations on how antipredator behavior changes over time and the extent to which these responses are affected by parental mobbing behavior are lacking.

Ravens are good candidates to fill these gaps in knowledge. Mobbing behavior has been previously studied in sub-adult non-breeding ravens with a similar methodology to some of the above-mentioned studies (Swift and Marzluff 2015) and it has been shown to be plastic in relation to the social context. When group-housed ravens were presented with two masked humans, a neutral person and a ‘dangerous’ person carrying a dead raven, they quickly learned to discriminate between them and remembered the difference for the next four years (Blum et al. 2020). Individual differences in mobbing could be partly explained by the birds’ upbringing, as parent-raised ravens engaged in more predator-directed mobbing than hand-raised ravens, indicating the importance of early life environment for the development of antipredator behavior. Another source of individual variation in mobbing was based on social context and rank: dominant individuals scolded more than lower ranking ones when tested with the dangerous mask in their social group as compared to in isolation (Blum et al. 2022). So, sub-adult ravens can discriminate between a dangerous and a neutral human, their antipredator behavior is plastic in relation to rank and influenced by their early experiences. Still, we do not know much about the antipredator behavior of raven breeding pairs, nor do we know how it might be influenced by the presence of offspring of varying developmental stages. Ravens provide their offspring with bi-parental care up to a few months after fledgling (Heinrich 1999). In this period, the offspring are highly neophilic (Heinrich 1995) and apparently need their parents’ protection, as they might still be naïve to predators. Hence, the family phase might be critical for the development of appropriate antipredator behavior.

In this study, we investigated how developmental stage and social context influence antipredator behavior in families of captive ravens, emphasizing the cognitive and behavioral mechanisms underlying this dynamic. To elicit antipredator responses in a standardized way, we used a similar method described by Blum et al. (2022) and presented our subjects with a masked human holding a dead raven (hereafter referred to as ‘dangerous human’ DH). We tested each family at two stages of offspring development: shortly after fledging (May) and nearly before independence (June/July). We scored four antipredator behaviors (scold, approach, investigate, and hide) that can be placed on a continuum from behavior directed at the predator to behavior directed away from the predator. In addition, we also scored the behavior ‘ignore’, in case the offspring did not react to the predator at all. Our study was guided by two hypotheses related to the parents’ and the offsprings’ behavior.

### (H1) Parental hypothesis

The parents’ antipredator behavior is affected by the age of their offspring. Our reasoning here is that parents should put themselves at risk when the offspring is vulnerable and not yet capable of reacting appropriately to the DH themselves. We thus predict parents to scold and approach the DH more in the early test period as in the late period.

### (H2) Offspring hypothesis

The offspring’s antipredator behavior is dependent on their age. We expect that shortly after fledging, the offspring will be less mobile and aware of the danger and will either ignore the DH or show more behavior directed away from the predator. As they grow older, they will show more awareness of the DH presence and engage in more behaviors directed towards the predator such as scold, approach, and investigate. Given the short time between the test periods, we might find an increase in the variance of the offspring’s responses, possibly reflecting the emergence of consistent individual differences. On the other hand, if the parents all respond to the DH in a similar way, the offspring’s behavior could also become more homogenous as they learn from them.

## Methods

### Subjects and rearing conditions

Study subjects were a total of 48 captive ravens (32 offspring, 16 parents), divided in 12 families consisting of two parents and a variable number of offspring (between one and four individuals). The experiment was run in two years (2022 and 2023), with some of the parents being present in both years but with different offspring. One family in 2023 only had one parent because the male went missing before the offspring fledged, and another family in 2022 lost their only offspring in the period when we were running the experiment, so they only participated to the first half of the experiment (see Supplementary Table S1 for further detail on the subjects).

The families were housed in large outdoor aviaries, one family unit per aviary, at three locations: Konrad-Lorenz-Forschungsstelle (hereafter indicated as KLF), Forschungsstation Haidlhof (HH), and Tiergarten Schönbrunn (TGS, Zoo Vienna). The aviaries at KLF and HH are similar in size (80–100 m^2^) and located in close distance (within hearing range) but mostly out of sight from each other (see sketch in Supplementary Fig. S1). At both sites, we kept four breeding pairs. The aviary at TGS is the largest (approximately 160 m^2^) and housed one single pair. Each aviary had a nesting niche where the birds could also retreat and shelter from the elements. The ground substrate consisted of wood chips, rocks, sand, and soil. Branches, plants, and rocks provided enrichment and perching opportunities. The ravens were fed twice daily on a variable diet including meat, fruits, grains, and vegetables. Water was available ad libitum. All ravens were marked for identification with colored rings.

### Experimental procedure

The subjects were tested during the family phase in two test periods, resembling different developmental stages: (i) early period, when the offspring had just fledged and were still highly dependent on the parents (mean offspring age at first presentation: 51± 5 days), and (ii) late period, when the offspring was almost independent and ready to leave the family (mean offspring age at first presentation: 103±9 days). In each period, the families were tested two times in a row, with at least one day in between the two presentations (mean = 2 days, min = 1 day, max = 5 days) at the KLF and HH. Due to practical constrains, there was only one presentation per period at TGS.

**Fig. 1:**
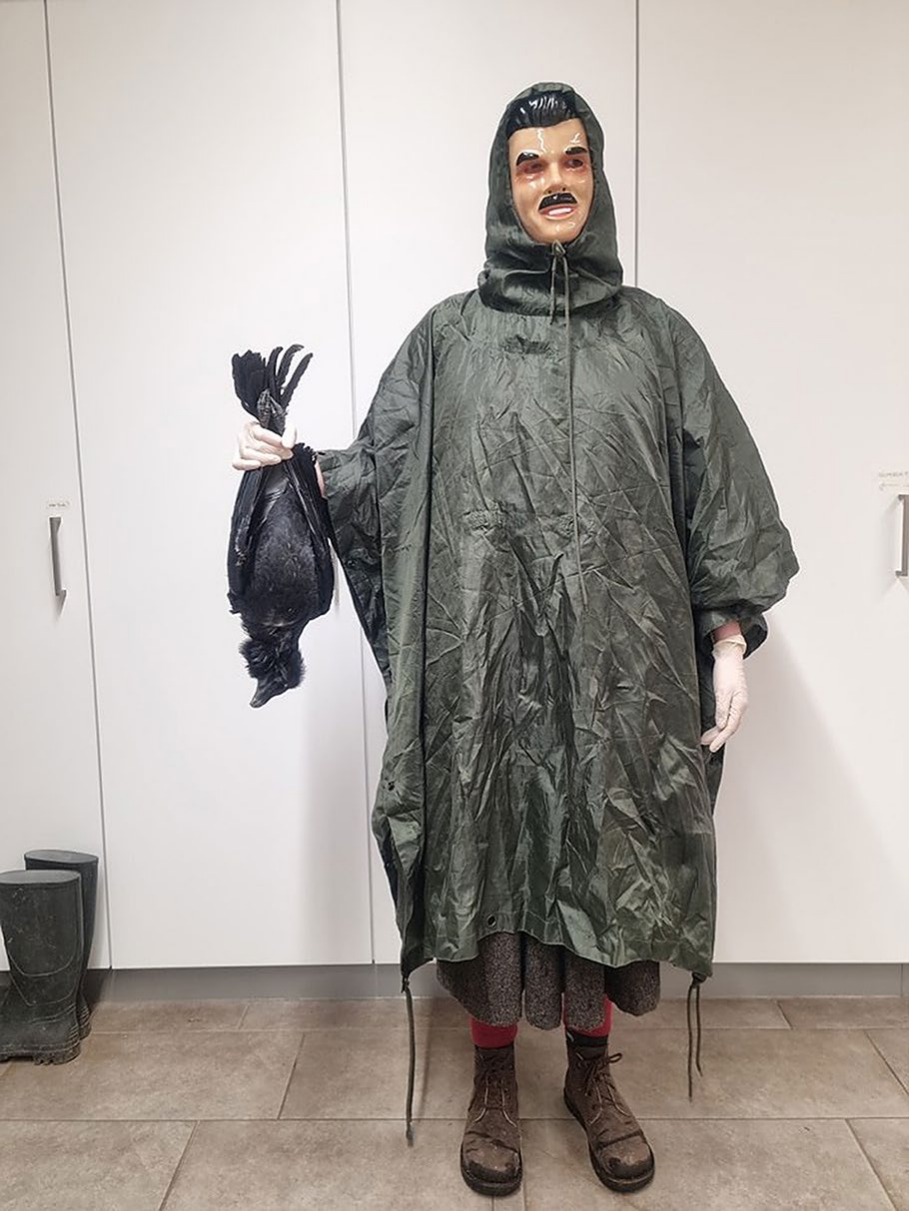
Picture of experimenter dressed as dangerous human (DH) and holding a dead raven

The test procedure was the same for each of the four presentations and for all locations. During each presentation, one person acted as a dangerous human (DH, Fig. 1) by wearing a mask with human features and standardized clothing (olive green hooded rain poncho, white surgical gloves), and holding a dead raven. The DH followed a standardized route that was approached from randomized directions to account for potential order effects, between the aviaries, passing in front of each aviary only once per presentation. The DH stopped at two positions in front of every aviary for two minutes each; the positions were about five meters apart and chosen to ensure that all subjects in the aviary had the chance of seeing the DH. During these stops, the DH moved the dangling dead raven in various positions.

We used different humans acting as DH, whereby the same person was used only for two trials. Various dead ravens were used, their bodies were intact and without external signs of injuries. They were wild ravens unfamiliar to the test subjects and sourced from the Cumberland Wildpark, after they were found dead in the wolf enclosure. Since the aviaries were within hearing range, we do not know if the birds affected each other by their vocalizations and to what extent. To control for this, the direction of the route taken by the DH was counterbalanced whenever possible, so that each time a different family would be the first to see the DH. Because of the small sample size, we could not test for this potential order effect, but we included the family ID as a random effect in our models. Most of the adult ravens were already familiar with the procedure because they were involved in previous antipredator experiments (Blum et al. 2022). The last time any of them was involved in an experiment with the DH was in February 2020 (see Supplementary Table S2 for details). All the offspring were naïve to the DH. In both periods, they were fledged and mobile, and regularly walking/flying around the aviary. All offspring were marked before fledging. Some of them reacted with loud alarm calls when taken out of the nest or otherwise handled, showing that they have the capacity to react to the presence of a heterospecific with loud vocalization.

### Video coding

The ravens were filmed with hand-held cameras while the DH was in front of their aviary. The visual and audio recordings of this cameras were then used to code every behavior, including vocalizations. The video analysis was performed using Solomon Coder (Péter, 2011). The behavior of the subjects in each aviary was coded from the time that the DH arrived in the first position in front of the aviary, to when the DH left the second position. The coding lasted for approximately four minutes per aviary (two minutes per position), but the amount of time spent in front of each aviary could vary slightly depending on the walking speed of the presenters; hence, we used a proportion of time spent in front of the aviaries for the analysis. We coded a total of five behaviors: scold, approach, investigate, ignore, and crouch (see supplementary table S3 for list of behaviors scored with description and type of data). Scolding is a distinctive alarm vocalization, representing the auditory component of mobbing, and it is only used in response to a DH. While the parameter approach represents the physical component of mobbing (which under field conditions might also include attacks). Both components feature an active response directed towards DH. Although they often go together in adults, we coded the two behaviors separately to see if either of the components were affected by presentation time and/or development stage. Investigate is a behavioral category indicating vigilance, and likely describes an active way of gathering information about the DH. Ignore refers to the lack of any obvious antipredator behavior. Crouch is the only behavior directed away from the DH. It likely refers to the birds’ attempts to hide and make themselves less conspicuous to the DH. Since we tested captive animals, it was not possible for them to flee and escape from the DH. We did not include fleeing from the DH in the coded behavior because wild ravens tend to react to playbacks of alarm calls by either crouching or being allert, i.e. standing upright and scanning the environment, but hardly by flying away (Davídková et al. 2020; Gallego-Abenza et al. 2021). Moreover, similar responses were found in our previous studies on captive non-breeding ravens using the same experimental paradigm as in the current study (Blum et al. 2020). We also did not observe the offspring approaching the parents to seek protection.

### Statistical analysis

We conducted our Analysis in R (version 4.3.1; R Core Team 2023), using multiple generalized linear mixed models (Baayen 2008) using the function “glmmTMB” (“glmmTMB”; Mollie et al. 2017). Our response variables were durations of the behaviors scold, investigate, ignore, and crouch for adults (H1) and offspring (H2) respectively, each measured with 0.2 s precision. Because trial durations were not always exactly two minutes long but varied slightly due to walking speed of the presenters and layout and occupation of the aviaries, we calculated the response as a ratio of trial duration and scaled it between 0 and 1 (Smithson and Verkuilen 2006). For each of these resulting ratios we formulated a beta distribution model with logit link (McCullagh and Nelder 1989; Bolker 2008). We also investigated the frequency of approaches as additional response using a Poisson distribution.

For all resulting models we chose as test predictors the interaction of “Age Class” (offspring vs. parent) and “Period” (early vs. late) and the main effect of “Period”. Additionally, we included “Age Class” and “Station” (HH, KLF, TGS) as control variables. As random effects we included SubjectID, GroupID and Date, and all theoretically identifiable random slopes (Schielzeth and Forstmeier 2009; Barr et al. 2013). To identify random slopes we used the function fe.re.tab() (from the package “diagnostic_fcns” (Mundry 2023). We dummy coded and centered all categorical predictors to a mean of zero. In case this maximal model did not converge, we removed model terms one at a time, starting with the random slopes, until model convergence was achieved. Correlations among random slopes were originally included but had to be removed in all models due to convergence issues. We then ran an overdispersion test using the function “overdisp.test” from the package “diagnostic_fcns” (Mundry 2023), to test for overdispersion (dispersion parameter larger than 1), or underdispersion (dispersion parameter smaller than 1).

We then conducted a full null model comparison to investigate the overall effect of all our test predictors and avoid cryptic multiple testing (Forstmeier and Schielzeth 2011). We therefore compared this full model to a null model, lacking the interaction term and the main effect “Period”, but containing the main effect “Age Class” and all other fixed and random control predictors, using the function “anova()” with “test = Chisq”. In case the full model was significantly better, we continued, otherwise we ended our analysis.

In case the full model was significantly better, we tested for collinearity issues between our predictors by investigating the variance inflation factors of a linear model including all fixed effects, but lacking the interaction term and the random effects, using the function “vif” of the package “car” (version 3.1-2, Weisberg & Fox, 2011; Field 2005; Quinn & Keough 2002). We also visually investigated the best linear unbiased predictors (BLUPs) using the function “ranef.diagn.plot” (Mundry 2023) for normal or uniform distribution and an x-axis range of less than +/- 4 (Baayen 2008; Harrison et al. 2018).

In a second step, we investigated the variation of the response, by calculating the means for each of the levels of “Period” (early vs. late)” and “Age Class” (offspring vs. parent), and then calculating the absolute distance of each observation to that respective mean, followed by a square root transformation. We included this response in a gaussian distribution model following the same analytical approach as outlined above but could not keep this model due to problematic diagnostics. We managed to fit it to a beta distribution, by scaling the response between 0 and 1, and encountered no issues with model diagnostics.

We detected no collinearity issues with a maximal VIF of 1.003 (Zuur, 2010; Quinn & Keough, 2002). If visual investigation of BLUPs revealed issues, they were mentioned for each model in the results part. Dispersion parameters were provided for each model in the results part as well. For one model (“Crouch”), diagnostics were problematic, and we transformed our response to binary and ran a binomial model with logit link following the same procedure.

Finally, we investigated the model results using the function “summary”, which informed us on the differences between the levels of the categorical predictors. We also calculated p values for each predictor by dropping it from the model and comparing it to the full model using the drop1 function (Wei et al. 2012). This provides a p-value for the entire predictor, not for a comparison between two of its categorical levels. Specifically, when applied to the interaction term, a significant p-value was then used as justification to follow up with a post hoc test. We conducted our post hoc test using the function “emmeans” from the package “emmeans” (version 1.10.0; Lenth 2024), to calculate the contrasts in estimated marginal means. The provided p values were adjusted for multiple testing using the sidak method and the specific comparisons were manually set to contrast between age classes within periods (i.e. compare adult to chicks for early and late period).

The sample size comprised a total of 360 observations, collected from 48 subjects over 2 years.

## Results

### Scolding duration

**Fig. 2:**
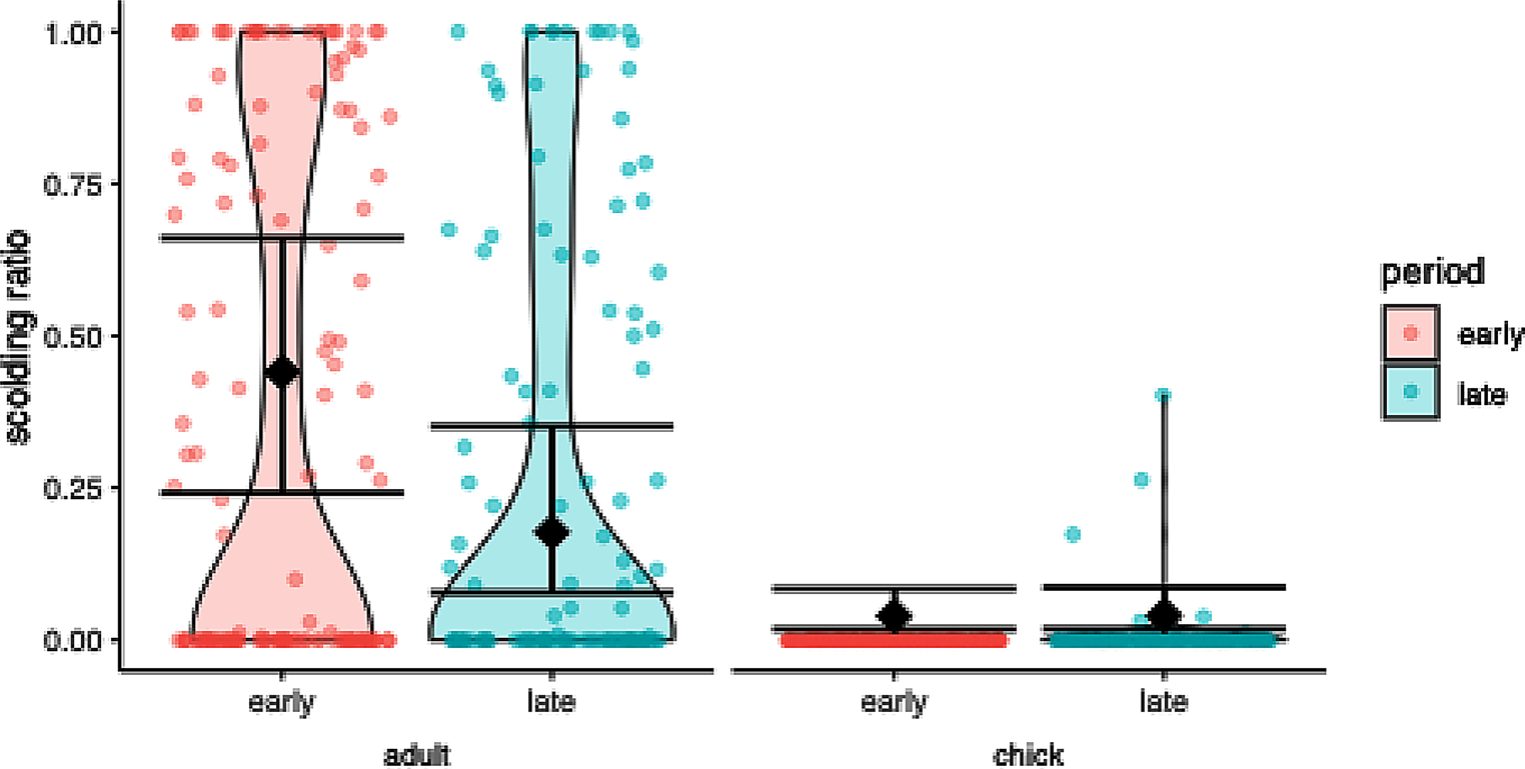
Scolding ratio per age class (offspring, parent) and test period (early, late) in response to a dangerous human in ravens. Raw data is depicted as violin plot with individual data points overlaid as scatter plot, model estimates and 95% confidence intervals as diamonds and error bars. Parents scolded less in the late period than in the early period, while offspring showed no significant difference between developmental periods

The full model, including the interaction between “Age Class” and “Period”, provided a significantly better fit than the reduced model without the interaction term and without the main effect of “Period” (χ2 = 9.155, df = 1, *P* = 0.034). This result indicates that the test predictors improve the model’s explanatory power, The full model was slightly under dispersed (dispersion parameter = 0.798), which could result in conservative estimates and increased likelihood of false negatives. Post hoc testing revealed that (i) parents scolded more in the early period compared to the late period, and (ii) offspring showed no difference in scolding behavior between the early and the late periods (Fig. 2; Table 1).

### Scolding variance

**Fig. 3:**
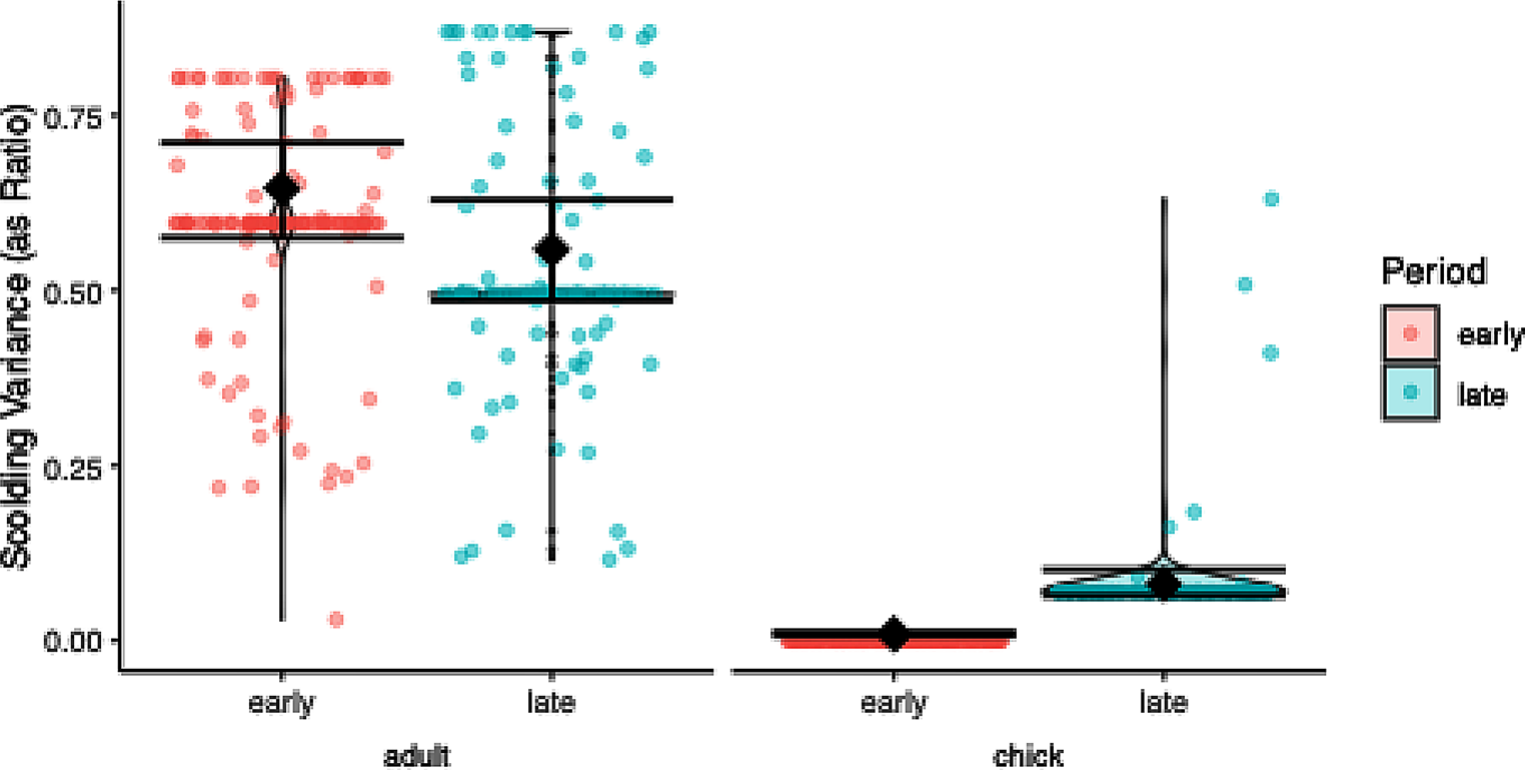
Scolding variance per age class (offspring, parent) and test period (early, late) in response to a dangerous human in ravens. Raw data is depicted as violin plot with individual data points overlaid as scatter plot, model estimates and 95% confidence intervals as diamonds and error bars. Offsprings’ variation was larger in the late period than in the early period, while the variance between the adults showed no significant change between periods

The model comparison was significant (χ2 = 94.354, df = 1, *P* < 0.001) and the dispersion was good (dispersion parameter = 0.990). The significant interaction term justified post hoc testing, which revealed larger variance in the late period than in the early period for the offspring (Fig. 3; Table 1).

### Approach frequency

**Fig. 4:**
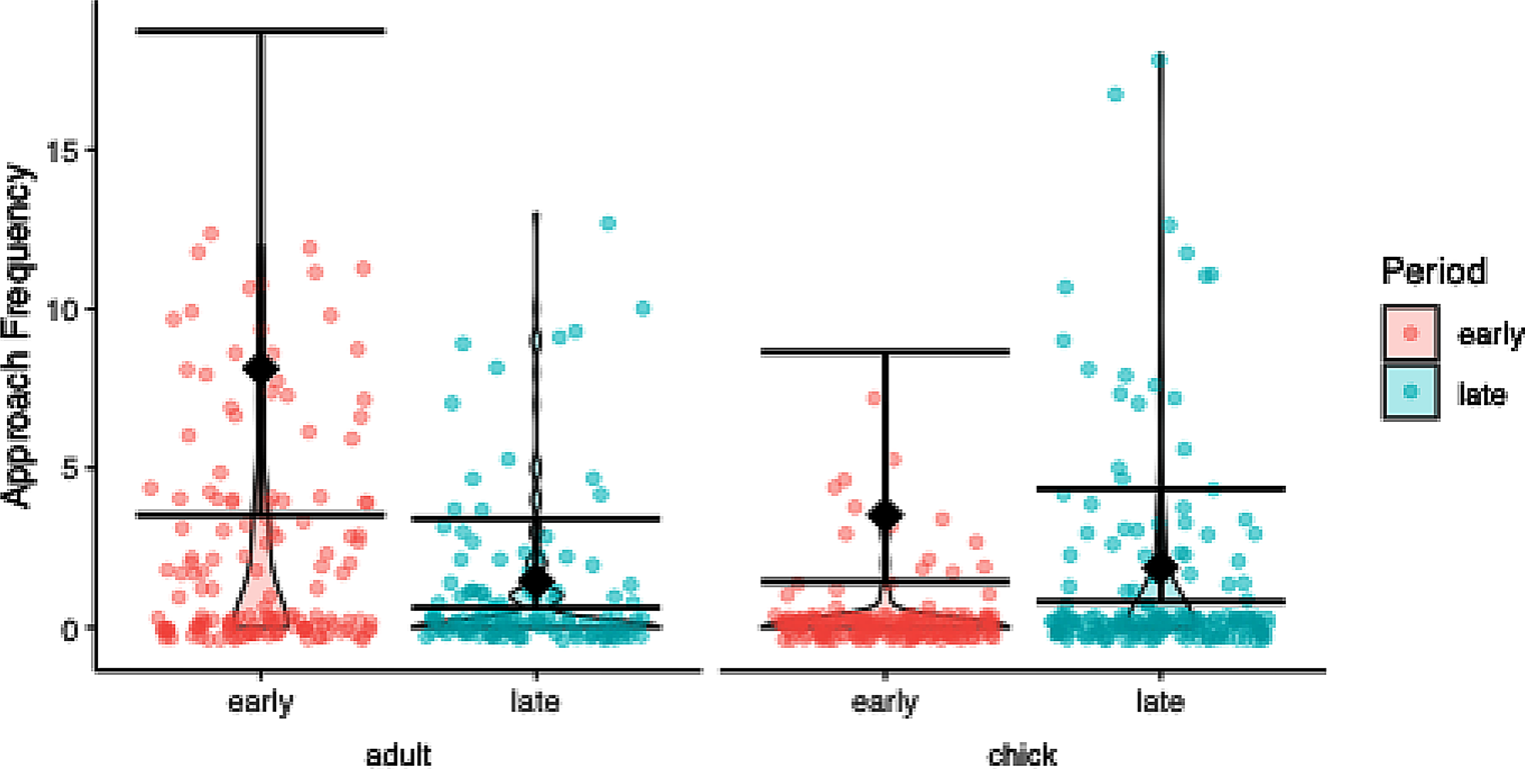
Approach frequency per age class (offspring, parent) and test period (early, late) in response to a dangerous human in ravens. Raw data is depicted as violin plot with individual data points overlaid as scatter plot, model estimates and 95% confidence intervals as diamonds and error bars. Parents approached less in the late period than in the early period, while offspring showed no significant difference between developmental periods

The model comparison was significant (χ2 = 36.801, df = 1, *P* < 0.001) and the dispersion was good (dispersion parameter = 0.986). The significant interaction term justified post hoc testing, which showed significantly more approaches for parents in early period compared to the late period (Fig. 4; Table 1).

### Approach variance

**Fig. 5:**
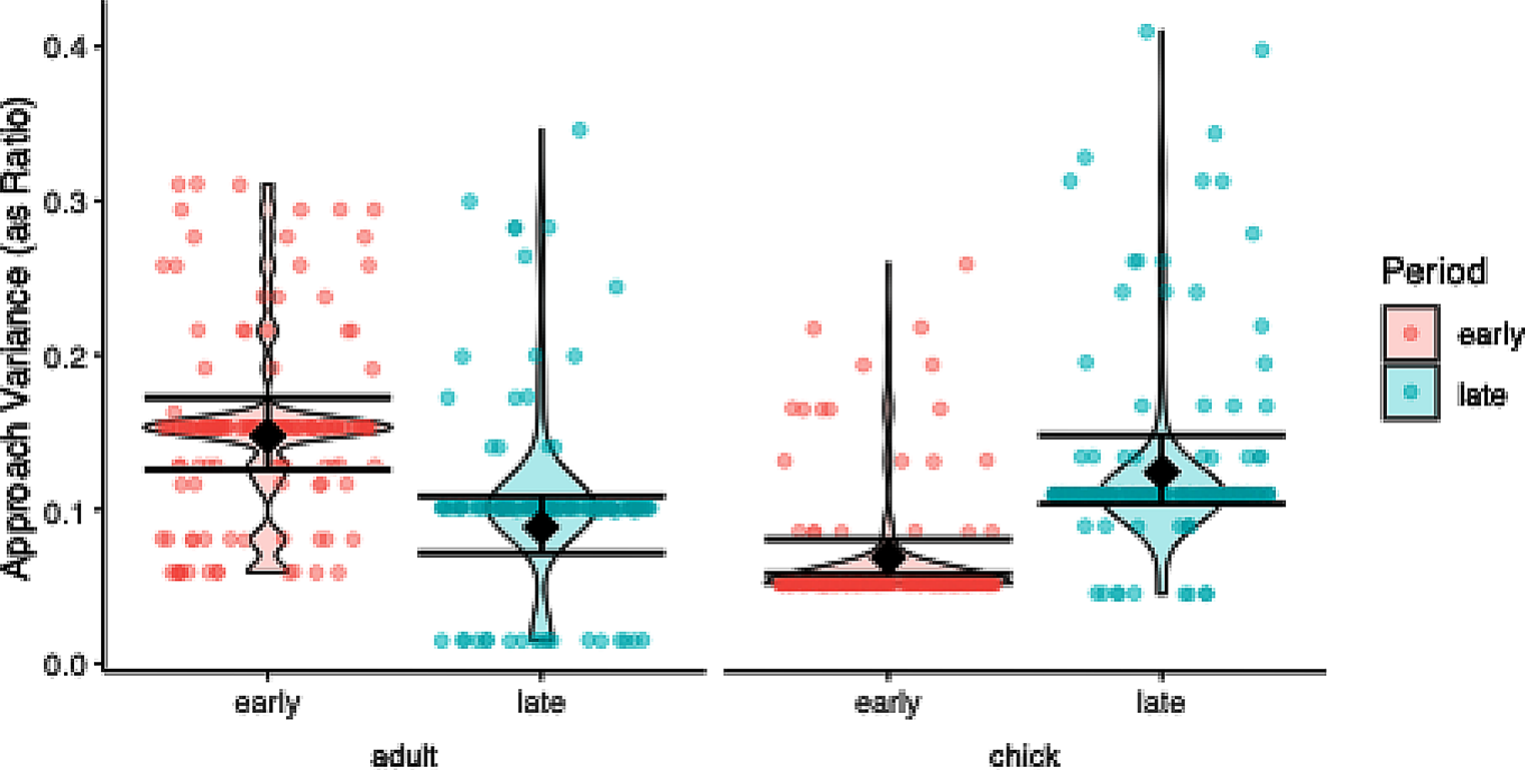
Approach variance per age (offspring, parent) and period (early, late) in response to a dangerous human in ravens. Raw data is depicted as violin plot with individual data points overlaid as scatter plot, model estimates and 95% confidence intervals as diamonds and error bars. Offsprings’ variation was larger in the late period than in the early period, while the variance between the adults was smaller in the late period compared to the early period

The model comparison was significant (χ2 = 34.816, df = 1, *P* < 0.001) and the dispersion was acceptable (dispersion parameter = 1.187). The significant interaction term justified post hoc testing, which showed: (i) significantly lower variation for adults in late period compared to the early period (ii) significantly higher variation for offspring in late period compared to the early period (Fig. 5; Table 1).

### Investigate duration

The model comparison was not significant (χ2 = 4.522, df = 1, *P* = 0.104), we therefore halted the analysis (see Supplementary Fig. S2 for plot of the raw data).

### Ignore duration

**Fig. 6:**
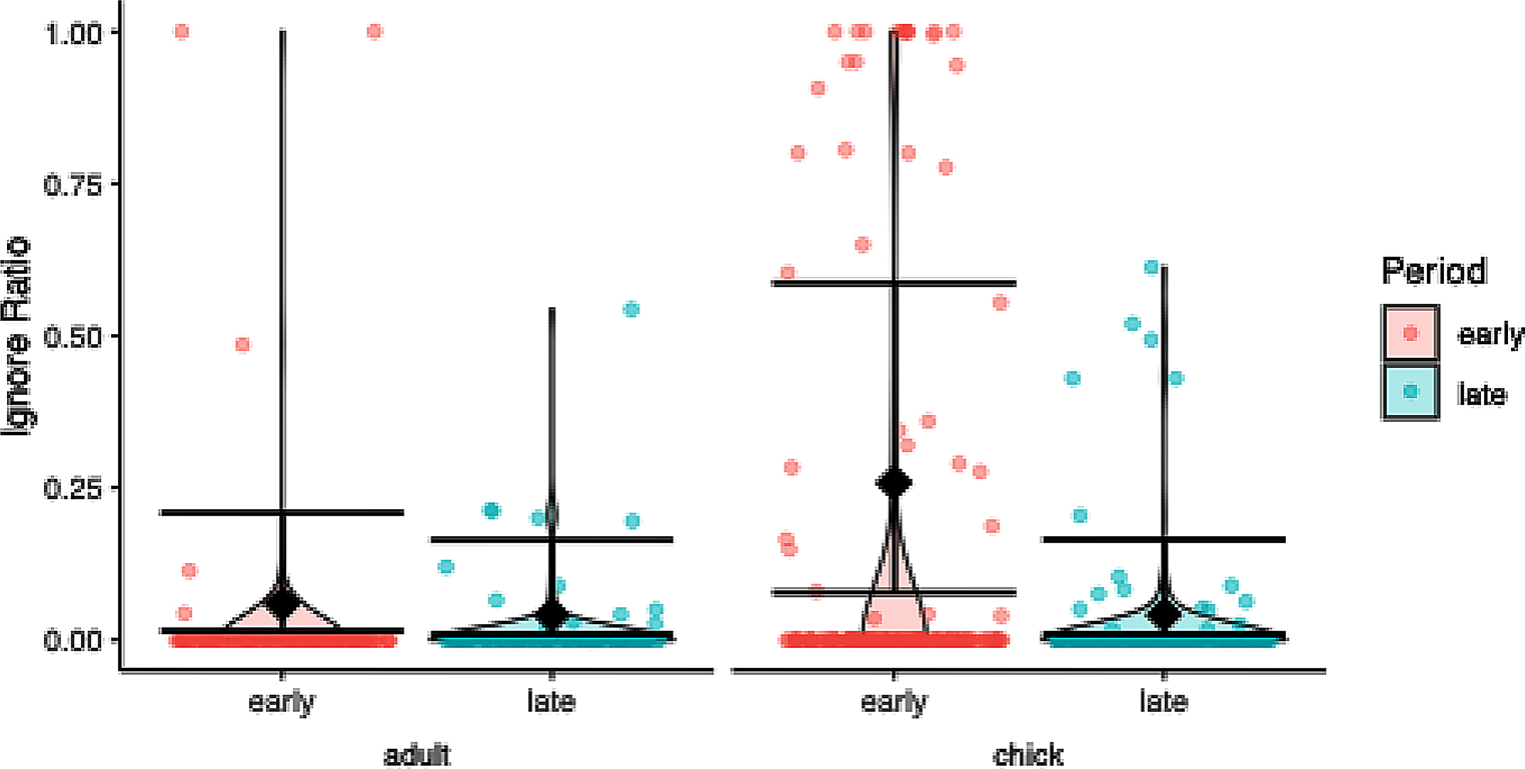
Ignore ratio per age class (offspring, parent) and test period (early, late) in response to a dangerous human in ravens. Raw data is depicted as violin plot with individual data points overlaid as scatter plot, model estimates and 95% confidence intervals as diamonds and error bars. Offspring ignored less in the late period than in the early period, while adults showed no significant difference between periods

Model comparison was significant (χ2 = 7.934, df = 1, *P* = 0.018), but the model was under dispersed (dispersion parameter = 0.816) leading to potentially conservative results. The significant interaction term justified post hoc testing, which found offspring ignored more in the early period (Fig. 6; Table 1).

### Ignore variance

**Fig. 7:**
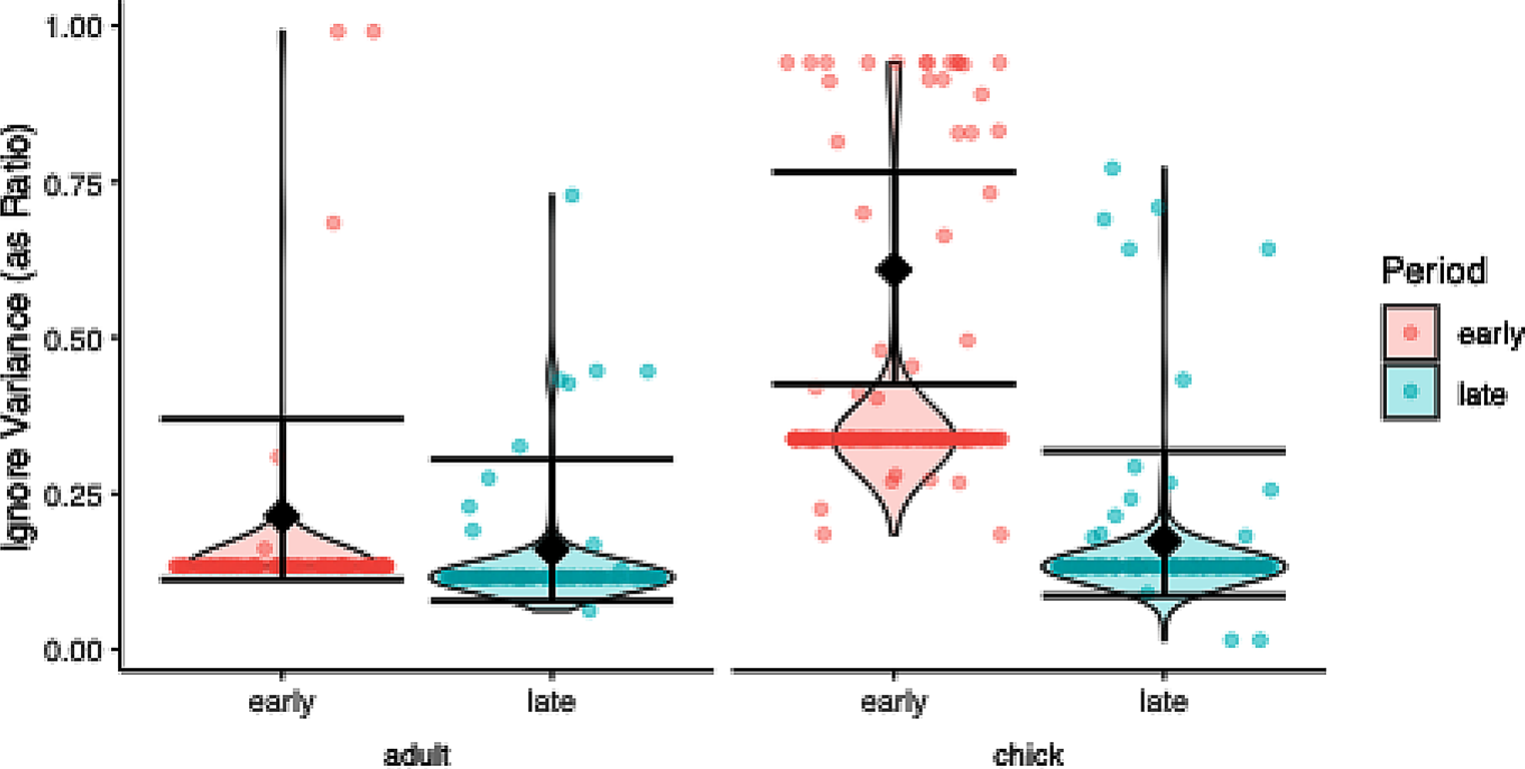
Ignore variance per age class (offspring, parent) and test period (early, late) in response to a dangerous human in ravens. Raw data is depicted as violin plot with individual data points overlaid as scatter plot, model estimates and 95% confidence intervals as diamonds and error bars. Offsprings’ variation was smaller in the late period than in the early period, while the variance between the adults showed no significant change between periods

The model comparison was significant (χ2 = 25.822, df = 1, *P* < 0.001), and the dispersion was good (dispersion parameter = 0.943). The significant interaction term justified post hoc testing, which revealed more variation in the early versus late period for offspring (Fig. 7; Table 1).

### Crouch duration

Diagnostics for this model were problematic. It was massively over dispersed (dispersion parameter = 4.744) and the range of the BLUPs was too large. We therefore transformed the response to binary and ran a binomial model, but this too suffered from the same issues. Due to the bad diagnostics and a non-significant model comparison (χ2 = 5.593, df = 1, *P* = 0.036) we stopped the analysis and provided a plot for the observations only (see Supplementary Fig. S3 for plot of the raw data).

**Table 1:** Post hoc contrasts for the interaction between age class (parent, offspring) and test period (early, late) for different anti-predator behaviors in response to a dangerous human (DH) in common ravens. The behaviors listed here are: scolding duration, scolding variance, approach frequency, approach variance, ignore duration, ignore variance

	Comparison	Estimate	SE	df	Z ratio	*P* value
Scolding duration	Adult early– Adult late	1.723	0.350	Inf	4.922	< 0.001
	Chick early– Chick late	0.628	0.383	Inf	1.639	0.192
Scolding variance	Adult early– Adult late	0.365	0.176	Inf	2.078	0.074
	Chick early– Chick late	-2.234	0.151	Inf	-14.793	< 0.001
Approach frequency	Adult early– Adult late	1.723	0. 350	Inf	4.922	< 0.001
	Chick early– Chick late	0.628	0. 383	Inf	1.639	0. 1920
Approach variance	Adult early– Adult late	0.578	0. 136	Inf	4. 253	< 0.001
	Chick early– Chick late	-0. 652	0. 126	Inf	-5.175	< 0.001
Ignore duration	Adult early– Adult late	0. 386	0. 946	Inf	0.408	0. 8998
	Chick early– Chick late	2.068	0. 899	Inf	-2.301	0. 0423
Ignore variance	Adult early– Adult late	0. 351	0. 474	Inf	0. 739	0. 7083
	Chick early– Chick late	2. 001	0. 451	Inf	4.435	< 0. 001

## Discussion

We here investigated how developmental stage and social context influence antipredator behavior in captive raven families. We exposed parents and their offspring to a potentially dangerous human (DH) shortly after the offsprings’ fledging (early test period in May) and close to their independence (late test period in June/July) and tested the hypotheses that (i) the parents’ antipredator behavior decreases with offsprings’ age and (ii) the offsprings’ antipredator behavior as well as the variance in their responses increase with age. We found that adults mobbed significantly more in the early test period, whereas the offspring were less likely to ignore the predator and showed higher variation in mobbing elements in the late test period.

### Parent behavior

Our findings are well in line with the prediction of our hypothesis and indicate that parental investment in antipredator mobbing is reduced with the offsprings’ age. Indeed, young ravens are highly vulnerable to predation in the first months post-fledge (own unpublished field data), but they also rapidly improve in their motoric and cognitive abilities during this time (e.g. ontogeny studies on food caching and object permanence, Bugnyar et al., 2007; gaze following, Schloegl et al., 2007). Around independence of their offspring, parents might thus afford reducing their time spent mobbing during predator encounters. Under naturalistic conditions with real predators, lower mobbing activities would directly relate to a reduced risk of being exposed to, and possibly being insured by, the predator and would save time and energy for other activities such as foraging or socializing.

An alternative interpretation to the reduction of parental investment with offsprings’ age might be that adult ravens quickly habituated to the test procedure (i.e., DH with dead raven). However, conceptual considerations and empirical data speak against this possibility. From a functional point, rapid habituation to potential predators should be prevented, as they might successfully strike at any time. From a mechanistic point, the mobbing behavior of parents got ‘rewarded’ every trial (as DH leaves without causing any harm) and should therefore be strengthened over time (compare Knight and Temple 1986; Griffin 2004; Marzluff et al. 2010). Accordingly, Blum et al. (2020) showed in a previous study with a similar set-up that mobbing responses of ravens to DH increased over the first exposure trials and then remained stable over years. A similar pattern has been reported for other corvids (Marzluff et al. 2010; Lee et al., 2021, Davidson et al. 2015). Finally, we do not see any noticeable difference in our data between the two exposures per test period (within May and within June/July) but a clear reduction of mobbing between the test periods (between exposures in May and June/July; Supplementary Fig. S4). Altogether, this strongly speaks against the argument of habituation.

### Offspring behavior

As predicted, the offspring engage more with the predator as they grow older and become independent. The only significant effect across individuals concerned the parameter ignore, which was shown more frequently in the early than the late test period and could have multiple explanations. Given the strong reactions of the parents, it is unlikely that the offspring failed to notice the DH’s presence in the early test period. One possibility is that their underdeveloped motor skills at this stage limited their ability to actively engage, although this does not explain the absence of other antipredator behaviors such as crouching. Another explanation is that young ravens need to learn from their parents what constitutes a predator and how to respond appropriately. Similar patterns have been observed in other species; for instance, Carlson et al. (2020) found that fledgling blue tits did not show a full mobbing response when exposed to playbacks of conspecific and heterospecific mobbing calls, suggesting that the recognition of threat and appropriate behavioral responses may require social learning. However, unlike the playback-only context of that study, our experimental setup exposed the offspring to both the predator (dangerous human) and the active behavioral responses of their parents. This raises the possibility that visual observation of adult behavior in conjunction with the perceived threat might play a stronger role in shaping the development of antipredator responses in ravens. The decline in ignore behavior over time could indicate that the offspring are learning the relevance of the DH as a threat. Nonetheless, we did not observe a significant increase in active mobbing behaviors in the late test period, so we cannot draw firm conclusions about antipredator learning. It is possible that mobbing behaviors take longer to develop in this species or are rarely expressed by offspring in the family context. Further studies should extend the observation period and include tests in isolation to better assess learning and development processes.

What we do find, however, is a significant difference in the variance in the offsprings’ responses towards DH between the early and the late test period. Specifically, we see a significance decrease in variance in the ignore response, and a significant increase in the variance of the approach and scold responses, between the early and late periods. For the ignore behavior, since the duration of the behavior also significantly decreases at the group level, this might indicate a general diminishing of a maladaptive behavior as the offspring grow older (see above). For the approach and scold behaviors instead, we see an increase in variance, so an increase of individual differences between the offspring as they age. Even if there is no significant increase in these behaviors in the offspring at the group level, these individual differences indicate that some ravens start to engage in predator directed behaviors as they age while others do not. This variation may reflect learning, individual differences in physiological maturation, or the development of personality traits (Griffin 2004; Cabrera et al., 2021), with some individuals consistently exhibiting bolder responses to predators than others. Future research could explore these patterns more deeply by building a larger dataset through repeated testing of individual offspring in isolation. Such an approach would allow for the assessment of the stability of behavioral traits over time, their potential dependence on physical development, and the influence of parental behavior. Moreover, testing individuals in isolation would help to disentangle these effects from those of the social environments.

To summarize, we presented families of ravens with a dangerous human and found that parents mob less as their offspring grow older, while the offspring ignore the DH less as they age and show an increased between-individual variance to engage in mobbing. For future directions, it would be interesting to explore whether the higher mobbing rates observed in parents during the early period could be explained by the teaching paradigm, which is defined as an individual modifying its behavior in a way that incurs a cost to its own fitness in order to facilitate learning in a naive observer (Caro and Hauser 1992). To test this hypothesis, future studies should assess the offsprings’ response to the DH, in the absence of both the dead raven and their parents, to determine whether learning has occurred. The second result, the diminishing of mobbing in the late period could be a decrease in parents’ risk-taking behavior as the offspring become independent. As for the offspring, the diminishing of the ignore behavior could be interpreted as them learning that the DH is an important stimulus, but there is no proof of increased antipredator behavior on their part yet. Between-individual variation in scolding and approaching could be also the result of (individual and social) learning and potentially tied to the development of personality traits. These findings may be applicable across taxa, especially in social species where parental care and group dynamics play critical roles in shaping adaptive responses. Our results underscore the importance of considering both developmental stages and social contexts when investigating antipredator behavior.

## Electronic supplementary material

Below is the link to the electronic supplementary material.

Supplementary Material 1

Supplementary Material 2

Supplementary Material 3

## Data Availability

Data is provided within the supplementary information files.

## References

[CR1] Altmann SA (1956) Avian mobbing behavior and predator recognition. Condor 58:241–253. 10.2307/1364703

[CR2] Baayen RH (2008) Analyzing linguistic data. Cambridge University Press, Cambridge

[CR4] Barr DJ, Levy R, Scheepers C, Tily HJ (2013) Random effects structure for confirmatory hypothesis testing Keep it maximal. J Mem Lang, 68:255–278 10.1016/j.jml.2012.11.001 PMC3881361 24403724

[CR7] Blum CR, Fitch WT, Bugnyar T (2020) Rapid learning and Long-Term memory for dangerous humans in Ravens (Corvus corax). Front Psychol 11:581794. 10.3389/fpsyg.2020.581794 33192900 10.3389/fpsyg.2020.581794 PMC7609869

[CR6] Blum CR, Fitch WT, Bugnyar T (2022) Social dynamics impact scolding behaviour in captive groups of common Ravens (Corvus corax). Front Zool 19:32. 10.1186/s12983-022-00477-6 36503565 10.1186/s12983-022-00477-6 PMC9743665

[CR8] Bolker BM (2008) Ecological models and data in R. Princeton University Press, Princeton New Jersey

[CR10] Buitron D (1983a) Variability in the responses of Black-Billed magpies to natural predators. Behav 87:209–235. 10.1163/156853983X00435

[CR65] Bugnyar T, Stöwe M, Heinrich B (2007) The ontogeny of caching in ravens, Corvus corax. Animal Behaviour 74:757–767. 10.1016/j.anbehav.2006.08.019

[CR11] Buitron D (1983b) Variability in the responses of black-billed magpies to natural predators. Behaviour 87:209–236

[CR66] Cabrera D, Nilsson JR, Griffen BD (2021) The development of animal personality across ontogeny: a cross-species review. Animal Behaviour 173:137–144. 10.1016/j.anbehav.2021.01.003

[CR12] Carlson NV, Griesser M (2022) Mobbing in animals: A thorough review and proposed future directions. Advances in the study of behavior. Elsevier, pp 1–41. 10.1016/bs.asb.2022.01.003

[CR13] Carlson NV, Healy SD, Templeton CN (2020) Wild fledgling Tits do not mob in response to conspecific or heterospecific mobbing calls. Ibis 162:1024–1032. 10.1111/ibi.12754

[CR15] Caro TM (2005) Antipredator defenses in birds and mammals. University of Chicago Press

[CR14] Caro TM, Hauser MD (1992) Is there teaching in nonhuman animals?? Q Rev Biol 67:151–174. 10.1086/417553 1635977 10.1086/417553

[CR16] Clark CW (1994) Antipredator behavior and the asset-protection principle. Behav Ecol 5:159–170. 10.1093/beheco/5.2.159

[CR17] Cornell HN, Marzluff JM, Pecoraro S (2012) Social learning spreads knowledge about dangerous humans among American crows. Proc R Soc B 279:499–508. 10.1098/rspb.2011.0957 21715408 10.1098/rspb.2011.0957 PMC3234554

[CR18] Courter JR, Ritchison G (2010) Alarm calls of tufted Titmice convey information about predator size and threat. Behav Ecol 21:936–942. 10.1093/beheco/arq086

[CR19] Cunha FCRD, Fontenelle JCR, Griesser M (2017) Predation risk drives the expression of mobbing across bird species. Behav Ecol 28:1517–1523. 10.1093/beheco/arx111

[CR20] Curio E (1978) The adaptive significance of avian mobbing: I. Teleonomic hypotheses and predictions. Z Für Tierpsychologie 48:175–183. 10.1111/j.1439-0310.1978.tb00254.x

[CR21] Curio E, Ernst U, Vieth W (1978) Cultural transmission of enemy recognition: one function of mobbing. Science 202:899–901. 10.1126/science.202.4370.899 17752463 10.1126/science.202.4370.899

[CR22] Davídková M, Veselý P, Syrová M, Nácarová J, Bugnyar T (2020) Ravens respond to unfamiliar Corvid alarm calls. J Ornithol 161:967–975

[CR23] Davidson GL, Clayton NS, Thornton A (2015) Wild jackdaws, Corvus monedula, recognize individual humans and May respond to gaze direction with defensive behaviour. Anim Behav 108:17–24. 10.1016/j.anbehav.2015.07.010

[CR24] Eggers S, Griesser M, Ekman J (2005) Predator-induced plasticity in nest visitation rates in the Siberian Jay (Perisoreus infaustus). Behav Ecol 16:309–315. 10.1093/beheco/arh163

[CR25] Ellis JMS (2009) Anti-Predator signals as advertisements: evidence in White‐Throated Magpie‐Jays. Ethology 115:522–532. 10.1111/j.1439-0310.2009.01631.x

[CR26] Field A (2005) Discovering statistics using SPSS. Sage, London

[CR28] Forstmeier W, Schielzeth H (2011) Cryptic multiple hypotheses testing in linear models: overestimated effect sizes and the winner’s curse. Behav Ecol Sociobiol 65:47–55. 10.1007/s00265-010-1038-5 21297852 10.1007/s00265-010-1038-5 PMC3015194

[CR27] Francis AM, Hailman JP, Woolfenden GE (1989) Mobbing by Florida scrub jays: behaviour, sexual asymmetry, role of helpers and ontogeny. Anim Behav 38:795–816. 10.1016/S0003-3472(89)80112-5

[CR29] Gallego-Abenza M, Blum CR, Bugnyar T (2021) Who is crying wolf? Seasonal effect on anti-predator response to age-specific alarm-calls in common ravens, Corvus corax. Learn Behav. 10.3758/s13420-020-00455-0 33420703 10.3758/s13420-020-00455-0 PMC7979661

[CR30] Grabarczyk EE, Ritchison G (2015) Vocal responses of adult Eastern bluebirds (*Sialia sialis*) to potential nest predators and the behavioral responses of nestlings. Wilson J Ornithol 127:697–705. 10.1676/14-190.1

[CR31] Griesser M, Ekman J (2005) Nepotistic mobbing behaviour in the Siberian jay, Perisoreus infaustus. Anim Behav 69:345–352. 10.1016/j.anbehav.2004.05.013

[CR33] Griesser M, Suzuki TN (2016) Kinship modulates the attention of Naïve individuals to the mobbing behaviour of role models. Anim Behav 112:83–91. 10.1016/j.anbehav.2015.11.020

[CR32] Griesser M, Suzuki TN (2017) Naive juveniles are more likely to become breeders after witnessing predator mobbing. Am Nat 189:58–66. 10.1086/689477 28035889 10.1086/689477

[CR34] Griffin AS (2004) Social learning about predators: a review and prospectus. Learn Behav 32:131–140. 10.3758/BF03196014 15161148 10.3758/bf03196014

[CR35] Ha J, Lee K, Yang E, Kim W, Song H, Hwang I, Lee-Cruz L, Lee S, Jablonski P (2020) Experimental study of alarm calls of the Oriental tit (*Parus minor*) toward different predators and reactions they induce in nestlings. Ethology 126:610–619. 10.1111/eth.13012

[CR36] Harrison XA, Donaldson L, Correa-Cano ME, Evans J, Fisher DN, Goodwin CED et al (2018) A brief introduction to mixed effects modelling and multi-model inference in ecology. PeerJ [Internet] 2018(5):e4794 10.7717/peerj.4794 PMC5970551 29844961

[CR37] Heinrich B (1995) An experimental investigation of insight in common Ravens (Corvus corax). Auk 112:994–1003. 10.2307/4089030

[CR38] Heinrich B (1999) Planning to facilitate caching: possible suet cutting by a common Raven. Wilson Bull. 276–278

[CR39] Ives AR, Dobson AP (1987) Antipredator behavior and the population dynamics of simple Predator-Prey systems. Am Nat 130:431–447. 10.1086/284719

[CR40] Knight RL, Temple SA (1986) Why does intensity of avian nest defense increase during the nesting cycle?? Auk 103:318–327. 10.1093/auk/103.2.318

[CR41] Lee HN, Greggor AL, Masuda B, Swaisgood RR (2021) Anti-Predator vigilance as an Indicator of the costs and benefits of supplemental feeding in newly released ‘alalā (Corvus hawaiiensis). Front Conserv Sci 2:701490. 10.3389/fcosc.2021.701490

[CR42] Lenth R (2024) _emmeans: Estimated Marginal Means, aka Least-Squares Means_. R package version 1.10.0, <https://CRAN.R-project.org/package=emmeans

[CR43] Levey DJ, Londoño GA, Ungvari-Martin J, Hiersoux MR, Jankowski JE, Poulsen JR, Stracey CM, Robinson SK (2009) Urban mockingbirds quickly learn to identify individual humans. Proc. Natl. Acad. Sci. U.S.A. 106, 8959–8962. 10.1073/pnas.0811422106 10.1073/pnas.0811422106 PMC2690012 19451622

[CR44] Lima SL, and Lawrence M. Dill (1990) Behavioral decisions made under the risk of predation: a review and prospectus. Can J Zool 68:619–640

[CR45] Marzluff JM, Walls J, Cornell HN, Withey JC, Craig DP (2010) Lasting recognition of threatening people by wild American crows. Anim Behav 79:699–707. 10.1016/j.anbehav.2009.12.022

[CR46] McCullagh P, Nelder JA (1989) Generalized linear models. Chapman and Hall London

[CR48] Mollie E, Brooks K, Kristensen, Koen J, van Benthem A, Magnusson CW, Berg A, Nielsen HJ, Skaug M, Maechler, Bolker BM (2017) GlmmTMB balances speed and flexibility among packages for Zero-inflated generalized linear mixed modeling. R J 9(2):378–400. 10.32614/RJ-2017-066

[CR49] Montgomerie RD, Weatherhead PJ (1988) Risks and rewards of nest defence by parent birds. Q Rev Biol 63

[CR50] Mundry R (2023) Some R functions. 10.5281/zenodo.7670524. Zenodo

[CR63] Péter A (2011) An introduction to Solomon coder. presentation.

[CR51] Quinn GP, Keough MJ (2002) Experimental design and data analysis for biologists. Cambridge University Press, Cambridge, United Kingdom

[CR52] R Core Team (2023) _R: A Language and Environment for Statistical Computing_. R Foundation for Statistical Computing, Vienna, Austria. https://www.R-project.org/

[CR53] Schielzeth H, Forstmeier W (2009) Conclusions beyond support: overconfident estimates in mixed models Behav Ecololgy, 20:416–420 10.1093/beheco/arn145 PMC2657178 19461866

[CR64] Schloegl C, Kotrschal K, Bugnyar T (2007) Gaze following in common ravens, Corvus corax: ontogeny and habituation. Animal Behaviour 74:769–778. 10.1016/j.anbehav.2006.08.017

[CR55] Smithson M, Verkuilen J (2006) A better lemon squeezer? maximum-likelihood regression with beta-distributed dependent variables. Psychol Methods, 11:54–71 10.1037/1082-989X.11.1.54 16594767

[CR57] Suzuki TN (2011) Parental alarm calls Warn nestlings about different predatory threats. Curr Biol 21:R15–R16. 10.1016/j.cub.2010.11.027 21215927 10.1016/j.cub.2010.11.027

[CR58] Swift KN, Marzluff JM (2015) Wild American crows gather around their dead to learn about danger. Anim Behav 109:187–197. 10.1016/j.anbehav.2015.08.021

[CR59] Templeton CN, Greene E, Davis K (2005) Allometry of alarm calls: Black-Capped chickadees encode information about predator size. Science 308:1934–1937. 10.1126/science.1108841 15976305 10.1126/science.1108841

[CR60] Wei J, Carroll RJ, Harden KK, Wu G (2012) Comparisons of treatment means when factors do not interact in two-factorial studies. Amino Acids.;42(5):2031-5. doi: 10.1007/s00726-011-0924-0. Epub 2011 May 6. PMID: 21547361; PMCID: PMC3199378 10.1007/s00726-011-0924-0 PMC3199378 21547361

[CR61] Weisberg S, Fox J, An (2011) R companion to applied regression [Internet]. Thousand Oaks: Sage; [cited 2020 Aug 28]. https://experts.umn.edu/en/publications/an-r-companion-to-applied-regression

[CR62] Zuur AF, Ieno EN, Elphick CS (2010) A protocol for data exploration to avoid common statistical problems. Methods Ecol Evol [Internet] 1(1):3–14. 10.1111/j.2041-210X.2009.00001.x

