# Supplementary material for: When to mob? plasticity of antipredator behavior in common ravens’ families (Corvus corax) across offspring development
Source: Anim Cogn. 2025 Jul 3;28(1):55. doi: 10.1007/s10071-025-01976-9 (PMC12226649; doi:10.1007/s10071-025-01976-9)
Supplement: Supplementary file 3 — Supplementary Material 3 [file 10071_2025_1976_MOESM3_ESM.docx]

**Supplementary materials**

| Subjects | Age class | Family | Test year | Location | Number of presentations 2022 | Number of presentations 2023 |
| --- | --- | --- | --- | --- | --- | --- |
| Heidi | Adult | HeTo | 2022, 2023 | KLF | 4 | 4 |
| Tom | Adult | HeTo | 2022, 2023 | KLF | 4 | 4 |
| Cobalt | Offspring | HeTo | 2022 | KLF | 4 |  |
| Indigo | Offspring | HeTo | 2022 | KLF | 4 |  |
| Navy | Offspring | HeTo | 2022 | KLF | 4 |  |
| Suzuki | Offspring | HeTo | 2023 | KLF |  | 4 |
| Yamaha | Offspring | HeTo | 2023 | KLF |  | 4 |
| Ducati | Offspring | HeTo | 2023 | KLF |  | 4 |
| Moritz | Adult | MoHa | 2023 | KLF |  | 4 |
| Harriet | Adult | MoHa | 2023 | KLF |  | 4 |
| Tukey | Offspring | MoHa | 2023 | KLF |  | 4 |
| Paul | Adult | PaAr | 2023 | KLF |  | 4 |
| Aramis | Adult | PaAr | 2023 | KLF |  | 4 |
| Alisio | Offspring | PaAr | 2023 | KLF |  | 4 |
| Bora | Offspring | PaAr | 2023 | KLF |  | 4 |
| Mistral | Offspring | PaAr | 2023 | KLF |  | 4 |
| Joey | Adult | JoRo | 2022 | HH | 4 |  |
| Rocky | Adult | JoRo | 2022 | HH | 4 |  |
| Bristle | Offspring | JoRo | 2022 | HH | 4 |  |
| Downy | Offspring | JoRo | 2022 | HH | 4 |  |
| Plume | Offspring | JoRo | 2022 | HH | 4 |  |
| Kay | Adult | KaJa | 2022, 2023 | HH | 4 |  |
| Janis | Adult | KaJa | 2022, 2023 | HH | 4 |  |
| Chowder | Offspring | KaJa | 2022 | HH | 4 |  |
| Gazpacho | Offspring | KaJa | 2022 | HH | 4 |  |
| Miso | Offspring | KaJa | 2022 | HH | 4 |  |
| Pho | Offspring | KaJa | 2022 | HH | 4 |  |
| Stiegl | Offspring | KaJa | 2023 | HH |  |  |
| Nobel | Adult | NoGe, No | 2022,  2023 | HH | 2 | 4 |
| George | Adult | NoGe | 2022 | HH | 2 |  |
| Ani | Offspring | NoGe | 2022 | HH | 2 |  |
| Arlanda | Offspring | No | 2023 | HH |  | 4 |
| Skansen | Offspring | No | 2023 | HH |  | 4 |
| Spanga | Offspring | No | 2023 | HH |  | 4 |
| Romi | Adult | RoHe | 2023 | HH |  | 4 |
| Heinz | Adult | RoHe | 2023 | HH |  | 4 |
| Blackbird | Offspring | RoHe | 2023 | HH |  | 4 |
| Boeing | Offspring | RoHe | 2023 | HH |  | 4 |
| Jumbo | Offspring | RoHe | 2023 | HH |  | 4 |
| Spitfire | Offspring | RoHe | 2023 | HH |  | 4 |
| Rufus | Adult | RuMu | 2022,  2023 | TGS | 2 | 2 |
| Munia | Adult | RuMu | 2022,  2023 | TGS | 2 | 2 |
| Rhaegal | Offspring | RuMu | 2022 | TGS | 2 |  |
| Smaug | Offspring | RuMu | 2022 | TGS | 2 |  |
| Peanut | Offspring | RuMu | 2023 | TGS |  | 2 |
| Walnut | Offspring | RuMu | 2023 | TGS |  | 2 |
| Almond | Offspring | RuMu | 2023 | TGS |  | 2 |
| Pistachio | Offspring | RuMu | 2023 | TGS |  | 2 |

**Supplementary Table S1** List of individuals tested with information about their age class (adult, offspring), the family they belong to (combination of the first two letters of the parent’ names), the aviary location, the year they were tested in, and the number of presentations per year

| Subjects | Last presentation DH with dead raven | Last presentation DH without dead raven |
| --- | --- | --- |
| Heidi | 11.11.11 | 14.11.13 |
| Munia | 02.06.18 | 18.08.18 |
| Rufus | 02.06.18 | 18.08.18 |
| Aramis | 10.03.20 | 25.02.20 |
| George | 10.03.20 | 25.02.20 |
| Harriet | 10.03.20 | 25.02.20 |
| Heinz | 10.03.20 | 25.02.20 |
| Janis | 10.03.20 | 25.02.20 |
| Joey | 10.03.20 | 25.02.20 |
| Kai | 10.03.20 | 25.02.20 |
| Nobel | 10.03.20 | 25.02.20 |
| Paul | 10.03.20 | 25.02.20 |
| Rocky | 10.03.20 | 25.02.20 |
| Romi | 10.03.20 | 25.02.20 |
| Moritz | NA | 13.07.19 |
| Tom | NA | 21.10.15 |

**Supplementary Table S2** List of subjects with last exposure to ‘DH with dead raven’ and ‘DH without dead raven’ in previous experiments

| Behaviors | Description | Type of data |
| --- | --- | --- |
| Scolding | Alarm call directed at the DH | Duration |
| Approach | Change of position that shortens the distance between the subject and the DH | Frequency |
| Investigate | Observation of the surrounding with elongated neck and without approach (information seeking) | Duration |
| Ignore | Continue to engage in baseline behavior that is not directed at the DH (preening, playing, eating) | Duration |
| Crouch | Neck tucked between the shoulders, fluffed feathers | Duration |

**Supplementary Table S3** List of behaviors scored with description and type of data

**Supplementary Fig. S1** Simplified sketch of the position of the aviaries at the HH research station (A) and KLF research station (B). The dotted lines represent an example of the path the DH would take during a presentation. The dots represent the spot where the DH would stop and face the aviary

**
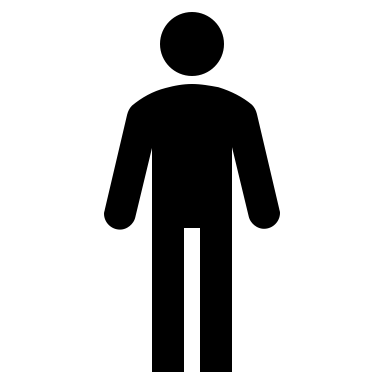
**

**
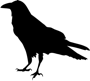

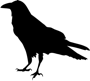
**

**
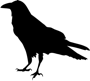

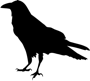
**

**
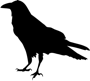

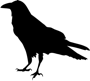
**

**
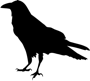

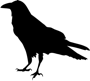
**

**
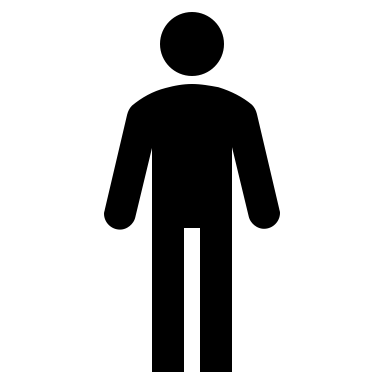
**

**A**


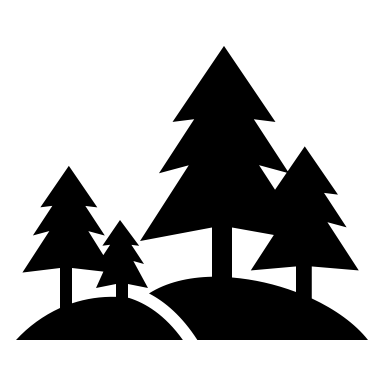


**
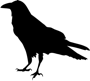

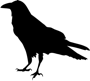
**

**
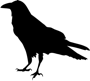

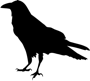
**

**
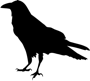

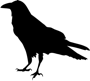
**


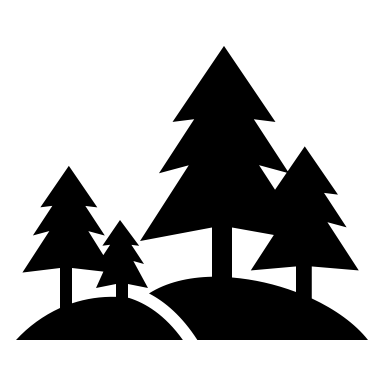


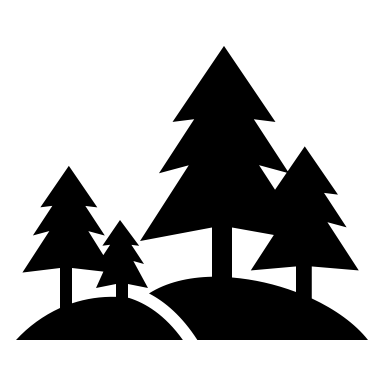


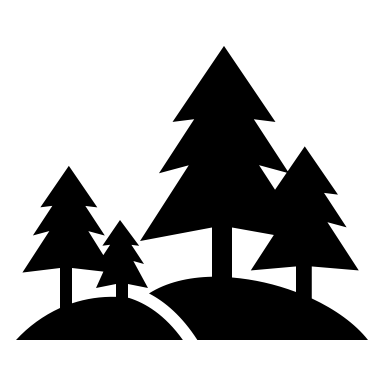


**
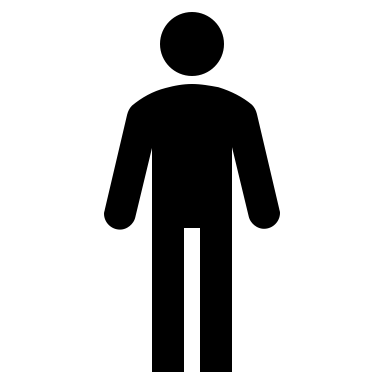
**

**
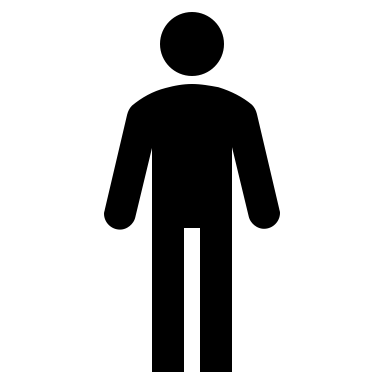
**

**B**


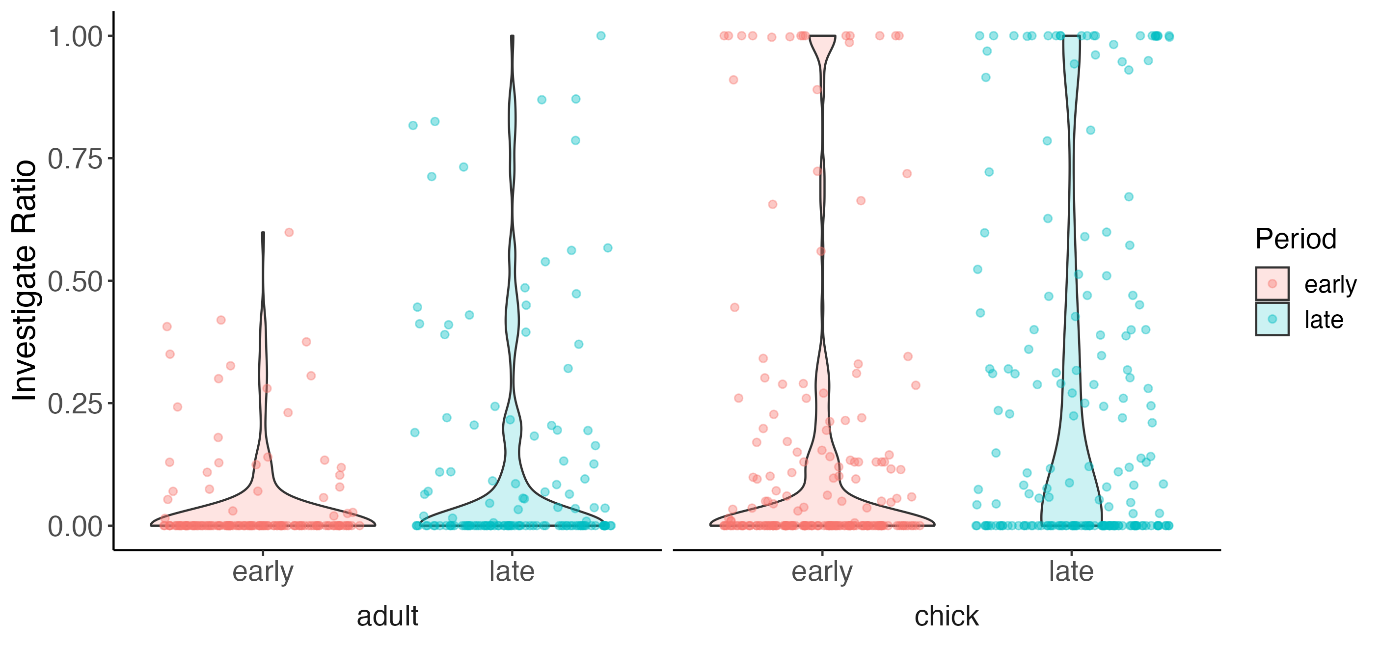


**Supplementary Fig. S2** Investigate ratio per age (offspring, parent) and period (early, late) in response to a dangerous human in ravens. Raw data is depicted as violin plot with individual data points overlaid as scatter plot. Model comparison was not significant

**Supplementary Fig. S3** Crouch ratio per age (offspring, parent) and period (early, late) in response to a dangerous human in ravens. Raw data is depicted as violin plot with individual data points overlaid as scatter plot. Model comparison was not significant

**
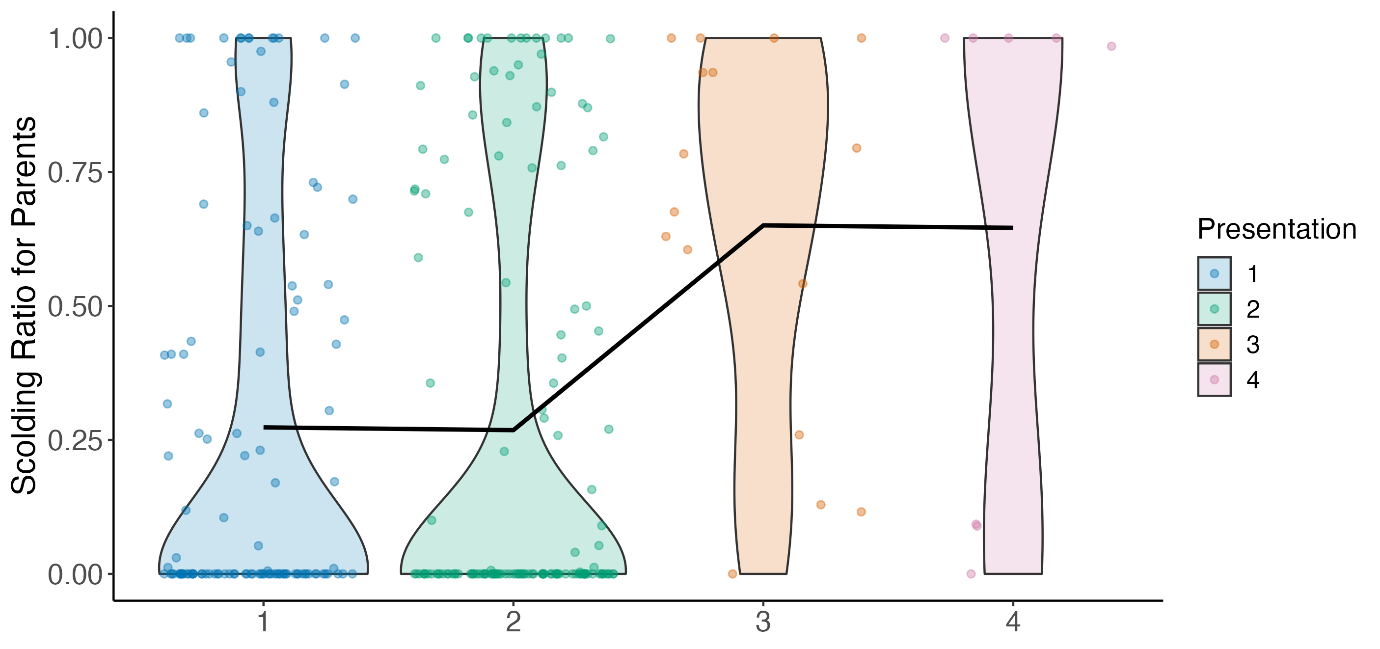
**

**Supplementary Fig. S4** Scolding ratio for parents per presentation (1, 2, 3, 4). Raw data is depicted as violin plot with individual data points overlaid as scatter plot. The black line connects the average response for each presentation
